# Relationship of Lumbar Lordosis With Non-specific Acute Low Back Pain

**DOI:** 10.7759/cureus.72453

**Published:** 2024-10-26

**Authors:** Sudhir Singh, Vijay P Singh, Shubham Jain

**Affiliations:** 1 Orthopedics, Teerthanker Mahaveer Medical College and Research Centre, Moradabad, IND; 2 Radiology, Teerthanker Mahaveer Medical College and Research Centre, Moradabad, IND

**Keywords:** acute low back pain, lumbar lordosis, lumbar lordotic angle, lumbosacral angle, non-specific, spino-pelvic parameter

## Abstract

Background: Low back pain (LBP) is a common health condition with an estimated prevalence of 42-83% in India. Clinicians usually measure lumbar lordotic angle (LLA) and lumbosacral angle (LSA) in sagittal radiographs even though the normal range of lordosis has not yet been agreed upon. Hence, the radiographic measurement of these angles needs to be re-evaluated. We aimed to study the difference in LLA and LSA in those with and without non-specific acute LBP and to analyze the correlation and association of confounding factors like age, gender, BMI, and pain severity with LLA and LSA.

Methods: LLA and LSA in those with and those without non-specific acute LBP in 200 individuals were recorded and analyzed statistically.

Results: In age, gender, and BMI-matched groups, the LSA in the cases group (34.44 ± 5.93) was significantly less than in controls (36.9 ± 6.8) (p = 0.007). LLA in the non-specific acute LBP group (50.51 ± 8.78) and those without non-specific acute LBP (50.05 ± 9.86) was statistically similar (p = 0.727). LSA was significantly less in patients than in healthy subjects. Both LLA and LSA were not associated withback pain and showed a weak or very weak correlation with age, gender, BMI, and severity of pain in both groups.

Conclusion: Lumbar lordosis didn’t show any association or correlation with age, gender, BMI, and VAS in non-specific acute LBP patients. Hence, measuring LSA and LLA in sagittal radiographs does not provide any additional information regarding the cause of pain in non-specific acute LBP patients.

## Introduction

Low back pain (LBP) is a common health condition characterized by pain and discomfort below the costal margin and above the inferior gluteal folds, with or without leg pain [1]. The estimated prevalence of LBP in India ranges between 42% and 83% [2-4]. LBP is defined as pain in the back from the level of the lowest rib down to the gluteal fold, with or without radiation into the legs [5]. Based on the duration of symptoms, back pain is labeled as acute back pain if it occurs for the first time in the patient’s life or if there has been a pain-free duration of six months after the previous episode and lasts for no more than six weeks [5]. However, pain for less than 12 weeks (three months) and less than four weeks of duration has also been labeled as acute back pain by others [6,7]. LBP is generally classified as either nonspecific or specific. Back pain is called nonspecific when there is no clear causal relationship between the symptoms and physical findings [5,7]. In clinical practice, about 80-90% of cases of LBP are nonspecific, that is, they have no clear patho-anatomical correlation [7-10].

A plethora of literature reports the influence of demographic factors (age, gender, race, BMI, ethnicity, and geographical location), occupational factors (jobs involving heavy lifting, repeated bending of the trunk, prolonged sitting, and physically demanding tasks), and psychosocial factors as risk factors for LBP. In the last two decades, the focus of researchers has been on sagittal spinopelvic parameters (posture), especially pelvic tilt, and lumbosacral angle (LSA) to assess lumbar lordosis on standing sagittal view radiographs of the spine [11]. Clinicians usually conduct a radiographic evaluation to evaluate these parameters. Lumbar lordosis and LSA are such parameters. The literature indicates that the amount of lordosis depends on factors such as age, gender, BMI, and ethnicity. The normal range of lordosis has not yet been agreed upon for gender, race, age, or geographical area. Therefore, the practice of radiographic measurement of the lumbar lordotic angle (LLA) and LSA in radiographs needs to be reevaluated [12]. In our study, we aimed to (1) determine the difference in LLA and LSA in patients with and without nonspecific acute LBP and (2) analyze the correlation and association of confounding factors such as age, gender, BMI, and pain severity with LLA and LSA.

## Materials and methods

This was a prospective case-control study, conducted from November 2022 to March 2024 in Teerthanker Mahaveer Medical College and Research Centre, Moradabad, India. The study proposal was approved by the College Research Committee and the Institutional Ethical Committee (TMU/IEC/2021-22/62). All procedures followed the ethical standards of the responsible committee on human experimentation (institutional and national) and of the World Medical Association Declaration of Helsinki (1975, revised 2013).

Sample size

The sample size was determined by a statistical formula \[n = \frac{z^2 \, p \, (100 - p)}{e^2}\]which suggested a minimum of 90 cases in each arm. We included 100 subjects each in the case and control groups.

Sample selection

A detailed history was taken, and a clinical examination was done for all patients who reported LBP. Those in whom LBP had occurred for the first time and had lasted for at least three months but not more than six months, with no clear patho-anatomical relation with the symptoms and physical findings, were diagnosed as having nonspecific acute LBP and were included in the study. We enrolled 100 patients with nonspecific acute LBP. We also enrolled 100 otherwise healthy individuals who had never experienced acute LBP. Study subjects of either gender and between 18 and 50 years old were selected. Patients were excluded if they indicated any of the following red flags: (a) significant trauma, (b) malignancy, (c) steroid use, (d) drug abuse, (e) immunocompromised state, (f) spinal or lower limb structural deformity, (g) inflammatory or infective spinal conditions, (h) neuromuscular conditions affecting the spine or lower limbs, (i) systemic disease with concomitant signs of infection, (j) cauda equina syndrome or radiculopathy, and (k) degenerative and osteoporotic spine. Subject selection was done by the first author.

Variables studied

LLA and LSA were the two parameters selected for evaluation on digital radiographs to assess lumbar lordosis. A lateral view of the lumbar spine was taken with the patient standing in a relaxed posture at a 90 cm distance from the X-ray tube. Radiographic parameters were measured by the second author, who was blinded for clinical findings, using Horos software version 3.0.

LSA was defined as the angle between the superior end plate of the first sacral vertebrae and a horizontal reference on sagittal imaging of the lumbosacral spine [13]. LLA was defined as the angle between the superior end plate of the L1 vertebra and the superior end plate of the S1 vertebral segment [14].

Statistical analysis

The categorical variables were displayed as numerical values and percentages (%). The quantitative data were displayed as the means ± standard deviation (SD). The normality of the data was assessed using the Shapiro-Wilk test. The subsequent statistical tests were utilized to analyze the outcomes.

The quantitative variables were compared using the independent t-test for two groups and ANOVA for more than two groups. The qualitative variables were evaluated using a chi-square test for comparison. Fisher’s exact test was employed if any cell had an expected value below 5. The Pearson correlation coefficient was used to correlate LSA and LLA with age, visual analog scale (VAS) score, and BMI. The final analysis was conducted using IBM SPSS Statistics for Windows, Version 25 (Released 2017; IBM Corp., Armonk, New York, United States). A p-value lower than 0.05 was used as the threshold for statistical significance.

## Results

There were 100 subjects each in the case group and control group.

Demographic profile

The age-wise distribution of subjects in each age group was similar. The case group consisted of 51 women and 49 men, whereas the control group consisted of 48 women and 52 men. The mean BMI of the case group was 26.61 ± 3.42 kg/m^2^, and that of the control group was 25.42 ± 5.37 kg/m^2^. Both groups were similar with respect to age (p = 0.903), gender (p = 0.671), and BMI (p = 0.063). Seventy subjects had moderate pain, and 30 had severe pain, with a mean VAS score of 5.99 ± 1.09 (Table 1).

**Table 1:** Demographic profile of subjects

		Cases (n = 100)	Controls (n = 100)	p-value
Age	18-30 years	26	31	0.434
	31-40 years	38	34	0.556
	41-50 years	36	35	0.883
	Mean ± SD	34.65 ± 9.2	34.81 ± 9.41	0.903
Gender	Female	51	48	0.671
	Male	49	52	
BMI	Underweight	0	6	0.029
	Normal BMI	30	45	0.041
	Overweight	55	28	0.0002
	Obese	15	21	0.358
	Mean ± SD	26.61 ± 3.42	25.42 ± 5.37	0.063
VAS	No pain (0)	0	100	-
	Mild pain (1-2)	0	0	
	Moderate pain (3-6)	70	0	
	Severe pain (7-10)	30	0	
	Mean ± SD	5.99 ± 1.09	-	-

LSA and LLA

The LSA was 34.44 ± 5.93 (men: 34.71 ± 6.47; women: 34.18 ± 5.41) in the case group and 36.9 ± 6.8 (men: 37.03 ± 6.05; women: 36.75 ± 7.59) in the control group, which was significantly lower than in the control group (p = 0.007; Table 2). LLA was 50.51 ± 8.78 (men: 49.71 ± 10.12; women: 51.28 ± 7.28) in the case group and 50.05 ± 9.86 (men: 50.71 ± 10.26; women: 49.32 ± 9.47) in the control group, which is similar (p = 0.727).

**Table 2:** Association of LSA with variables LSA: lumbosacral angle; VAS: visual analog scale

		Cases	Controls	p-value
Age	18-30 years	33.99 ± 6.95	35.36 ± 5.44	0.408
	31-40 years	34.35 ± 5.27	37.35 ± 7.81	0.058
	41-50 years	34.86 ± 5.94	37.82 ± 6.79	0.054
	p-value	0.849	0.307	-
Gender	Female	34.18 ± 5.41	36.75 ± 7.59	0.054
	Male	34.71 ± 6.47	37.03 ± 6.05	0.065
	p-value	0.659	0.838	-
BMI	Underweight	-	31.38 ± 2.33	-
	Normal BMI	32.46 ± 6.40	36.35 ±6.46	0.013
	Overweight	35.39 ± 5.79	37.28 ± 6.47	0.182
	Obese	34.89 ± 4.78	39.15 ± 7.98	0.074
	p-value	0.088	0.083	-
VAS	No pain (0)	-	-	-
	Mild pain (1-2)	-	-	-
	Moderate pain (3-6)	34.67 ± 6.15	-	-
	Severe pain (7-10)	33.9 ± 5.44	-	-
	p-value	0.557	-	

Association

LSA

Analysis of data showed that LSA is similar in all age subgroups in the case group (p = 0.849) and the control group (p = 0.307), indicating that they are not associated. Even in all age subgroups, the LSA was statistically similar in the case and control groups (18-28 years: p = 0.408; 29-39 years: p = 0.058; 40-50 years: p = 0.054), indicating no association of LSA with age.

LSA did not show any significant difference in values in either gender in the case group (p = 0.659) and the control group (p = 0.838). Even among women (p = 0.054) and men (p = 0.065), the LSA was similar, indicating no association with gender. LSA was similar in the case group (p = 0.088) and the control group (p = 0.083) in all BMI subcategories, thus showing no association with BMI. However, in the normal subcategory of BMI, LSA was significantly lower in the case group (p = 0.013). LSA was similar in the moderate and severe pain subgroup in the case group, showing no association with the severity of pain (VAS: p = 0.557; Table 2).

LLA

In our study, LLA showed no significant difference between the case and control groups as a whole (case: p = 0.799; control: p = 0.828) and between different age subgroups (18-28 years: p = 0.941; 29-39 years: p = 0.94; 40-50 years: p = 0.441), indicating no association of LLA with age. LLA was similar in either gender in both the case group (p = 0.377) and control group (p = 0.484). In addition, LLA was similar in men (p = 0.251) and women (p = 0.621) in both case and control groups (Table 3). LLA in all subcategories of BMI in the case and control groups was similar (case: p = 0.488; control: p = 0.104). Within the subcategories of BMI, there was no difference in LLA (normal: p = 0.578, overweight: p = 0.230, and obese: p = 0.310), showing no association. LLA was similar in moderate and severe pain subgroups in the case group, showing no association (p = 0.448; Table 3).

**Table 3:** Association of LLA with variables LSA: lumbosacral angle; VAS: visual analog scale

		Cases	Controls	p-value
Age	18-30 years	50.71 ± 8.29	50.89 ± 10.18	0.941
	31-40 years	49.78 ± 9.5	49.94 ± 9.29	0.941
	41-50 years	51.13 ± 8.5	49.39 ± 10.33	0.441
	p-value	0.799	0.828	-
	Female	51.28 ± 7.28	49.32 ± 9.47	0.251
Gender	Male	49.71 ± 10.12	50.71 ± 10.26	0.621
	p-value	0.377	0.484	-
	Underweight	-	42 ± 8.78	-
BMI	Normal	49.37 ± 9.2	50.5 ± 8.22	0.578
	Overweight	51.52 ± 8.69	48.95 ± 10.03	0.230
	Obese	49.09 ± 8.32	52.83 ± 12.16	0.310
	p-value	0.448	0.104	-
	No pain (0)	-	-	-
VAS	Mild pain (1-2)	-	-	-
	Moderate pain (3-6)	50.95 ± 8.48	-	
	Severe pain (7-10)	49.48 ± 9.51	-	
	p-value	0.448	-	

Correlation

LSA

Data analysis shows that LSA in the control group has a weak positive correlation with age (r = 0.127) and BMI (r = 0.279). LSA in the case group has a weak positive correlation with age (r = 0.074), BMI (r = 0.156), and VAS (r = 0.01; Table 4).

**Table 4:** Correlation of variables with LSA LSA: lumbosacral angle; VAS: visual analog scale

	Variables	Mean ± SD	Pearson correlation and coefficient value (r)	p-value
Controls	LSA	36.9 ± 6.8	0.127	0.207
	Age	34.8 ± 9.4		
	LSA	36.9 ± 6.8	0.279	0.005
	BMI	25.4 ± 5.3		
Cases	LSA	34.44 ± 5.93	0.074	0.466
	Age	34.65 ± 9.2		
	LSA	34.44 ± 5.93	0.156	0.122
	BMI	26.61 ± 3.42		
	LSA	34.44 ± 5.93	0.010	0.923
	VAS	5.99 ± 1.09		

LLA

LLA in the control group has a weak negative correlation with age (r = -0.061) and a weak positive correlation with BMI (r = 0.193). LLA in the case group shows a weak positive correlation with age (r = 0.055) and BMI (r = 0.006) and a weak negative correlation with VAS (r = -0.141; Table 5).

**Table 5:** Correlation of variables with LLA LLA: lumbar lordotic angle; VAS: visual analog scale

	Variables	Mean ± SD	Pearson correlation and coefficient value (r)	p-value
Controls	LLA	50.05 ± 9.86	-0.061	0.548
	Age	34.8 ± 9.4		
	LLA	50.05 ± 9.86	0.193	0.054
	BMI	25.42 ± 5.37		
Cases	LLA	50.51 ± 8.78	0.055	0.589
	Age	34.65 ± 9.2		
	LLA	50.51 ± 8.78	0.006	0.950
	BMI	26.61 ± 3.42		
	LLA	50.51 ± 8.78	-0.141	0.162
	VAS	5.99 ± 1.09		

## Discussion

Both the lumbar vertebrae and the sacrum contribute to developing the lordosis of the lumbar spine. Therefore, we studied the two important parameters of the lumbar spine, that is, LLA and LSA, on sagittal radiographs and analyzed the variation of these parameters in LBP patients and in normal healthy people. Moreover, we also attempted to find the effect of confounding factors such as age, gender, and BMI on these parameters. Different authors have labeled the LSA as the sacral horizontal angle, sacral angle, sacral inclination angle, sacral slope, or Ferguson’s angle [15].

Historically, a relationship between lumbar lordosis and LBP has been thought to be an important factor causing LBP. Measurement of these parameters of lordosis (LLA and LSA) serves as a guide in formulating a therapeutic regime for treating LBP [16]. After conducting a meta-analysis on this subject in 2017, Chun et al. [16] reported that an increased LSA is a known cause of LBP. However, this has been debated, and many systematic reviews and meta-analyses with conflicting conclusions stated that lumbar lordosis did not differ between subjects with and without LBP [12,17-21]. It has been reported that confounding factors such as age, sex, and BMI influence the degree of lordosis [22-24].

In our study, the study subjects with and without LBP were of similar age, gender, BMI, and pain duration, indicating that both the case and control groups were homogenous. LSA in patients with LBP was similar to that of a normal population in individual age groups and either gender or subcategories of BMI except in the normal subcategory that showed no association with age, gender, and BMI. However, LSA is significantly lower (p = 0.013) in patients with normal BMI (Table 3). It is uncertain that lower LSA values in the normal weight category are the cause or effect of back pain. First, if lower LSA values are either the cause or effect, they should be reflected in the overweight and obese categories as well. Second, we do not have the patients’ radiographs prior to the episode of pain to measure the LSA. Third, this might be because patients attempt to reduce the pain by posture compensation, resulting in a decreased LSA. However, due to increased body mass, overweight and obese people are unable to do posture compensation as the muscle strength becomes relatively insufficient. This reciprocal relationship between the sacral slope and the lumbar curvature as an essential component of overall sagittal alignment has previously been reported [25]. The author stated that when the sacral slope increases, the lordosis increases as well. When the sacral slope decreases, the lower arc of lordosis decreases or can flatten as the radius of curvature increases.

LLA in those with LBP was slightly higher than in the healthy population but not significant. LLA in both those with and without back pain was similar in all subgroups of age, BMI, and gender, showing no association with these confounding factors. Earlier reports have also stated no association of lordosis with back pain [12,17-18]. Further, LSA and LLA were similar in patients having moderate and severe pain, showing no association with the severity of pain (p = 0.557 and p = 0.448, respectively). Data analysis in our study shows that both LSA and LLA show only a weak correlation with age, gender, BMI, and severity of pain in both groups.

Over the last few decades, several countries have issued guidelines for the diagnosis and treatment of LBP, and there is an extensive repository of publications on clinical practice guidelines (CPGs) [7]. Earlier CPGs were primarily based on recommendations of the subject expert, but more evidence-based CPGs have recently emerged, which include implementation strategies for the management of nonspecific LBP [26-28]. Separate guidelines on the duration of acute LBP, subacute LBP, and chronic LBP have been recommended [28].

Our study has shown that there is no evidence that sagittal spino-pelvic parameter mensuration in radiographs provides any further insight to help in the diagnosis or treatment of LBP. In this study, lumbar lordosis has not shown any association or correlation with age, gender, body weight, or pain severity in LBP patients. Measuring LSA and LLA in radiographs has provided no additional or conclusive information regarding the cause of pain in acute LBP patients.

In India, the current CPGs for diagnosing and treating LBP patients are yet to be formulated and adopted by clinicians. The use of radiographs has an inherent risk of radiation exposure to the patient and thus should be avoided [29]. In the absence of red flags, radiography of the spine is unnecessary and not recommended and thus should be discouraged [7,12,29]. In addition, unnecessary radiography increases treatment costs and may cause treatment delays. The cause-and-effect relationship between LLA and LSA with LBP can only be elucidated by prospective longitudinal studies that relate current lumbar lordosis with future low back problems [16].

## Conclusions

The results show that LLA does not vary in those with and without non-specific acute LBP. LSA was significantly less in patients than in healthy subjects. Both LLA and LSA were not associated with back pain and showed a weak or very weak correlation with age, gender, BMI, and severity of pain in both groups.
